# Transcriptome Analysis Identifies Candidate Target Genes Involved in Glyphosate-Resistance Mechanism in *Lolium multiflorum*

**DOI:** 10.3390/plants9060685

**Published:** 2020-05-28

**Authors:** Joanei Cechin, Cristiano Piasecki, Daiane P. Benemann, Frederico S. Kremer, Vanessa Galli, Luciano C. Maia, Dirceu Agostinetto, Leandro Vargas

**Affiliations:** 1 Department of Crop Protection, Federal University of Pelotas, Pelotas, RS 96160-000, Brazil; jcechin@ufpel.edu.br (J.C.); daiane.benemann@ufpel.edu.br (D.P.B.); dirceu_agostinetto@ufpel.edu.br (D.A.); 2 Department of Plant Sciences, University of Tennessee, Knoxville, TN 37996, USA; 3 Center for Technological Development, Federal University of Pelotas (UFPel), Pelotas, RS 96010-610, Brazil; frederico.kremer@thrivedatascience.com (F.S.K.); vanessa.galli@ufpel.edu.br (V.G.); 4 Department of Plant Breeding, Federal University of Pelotas (UFPel), Pelotas, RS 96010-610, Brazil; luciano.maia@ufpel.edu.br; 5 Department of Weed Science, Brazilian Agricultural Research Corporation (Embrapa), Passo Fundo, RS 99050-970, Brazil; leandro.vargas@embrapa.br

**Keywords:** Italian ryegrass, next-generation sequencing, RNA-Seq, differential gene expression, resistance mechanism

## Abstract

Italian ryegrass (*Lolium multiflorum*; LOLMU) is one of the most troublesome weeds in temperate regions in the world. This weed species interfere with wheat, corn, rye, and oat, causing significant crop yield losses. This species has evolved glyphosate resistance, making it difficult to control. The mechanisms of glyphosate resistance are still unknown, and an understanding thereof will favor the development of new strategies of management. The present study is the first transcriptome study in LOLMU using glyphosate-resistant and -sensitive biotypes, aiming to identify and to provide a list of the candidate target genes related to glyphosate resistance mechanism. The transcriptome was assembled *de novo*, producing 87,433 contigs with an N50 of 740 bp and an average length of 575 bp. There were 92 and 54 up- and down-regulated genes, respectively, in the resistant biotype, while a total of 1683 were differentially expressed in the sensitive biotype in response to glyphosate treatment. We selected 14 highly induced genes and seven with repressed expression in the resistant biotype in response to glyphosate. Of these genes, a significant proportion were related to the plasma membrane, indicating that there is a barrier making it difficult for glyphosate to enter the cell.

## 1. Introduction

Weeds are the major problem in agricultural production worldwide because they cause high crop yield losses and economic damage. Italian ryegrass (*Lolium multiflorum* Lam.; LOLMU) is the most common annual weed found in fields in temperate climates [1,2]. LOLMU is a C_3_ annual grass that reproduces using seeds and is self-incompatible, allowing genetic diversity to evolve and adapt to a wide range of environments [2,3,4]. This grass grows vigorously, is highly competitive with crops, and generally is cultivated as pasture, favoring its high density [5,6]. LOLMU also interferes in wheat and corn, reducing crop yields by 60% and 48%, respectively [4,7].

In Brazilian agricultural fields in the past two decades, LOLMU has generally been controlled with glyphosate, a 5-enolpyruvylshikimate 3-phosphate synthase (EPSPS) inhibitor [8,9]. However, the repetitive application of glyphosate has selected glyphosate-resistant (GR) LOLMU populations. The first case of GR LOLMU in Brazil was reported in 2003, and it is now present over approximately 3.5 million hectares [6,9]. Moreover, it is estimated that the cost to control GR LOLMU is about 150% higher than to control glyphosate-sensitive (GS) plants, resulting in a significant economic impact [8].

Herbicide resistance is the result of weed evolution and the genetic variability of plants present in several environments. It can occur in two ways, involving either herbicide target-site resistance (TSR) or non-target-site resistance mechanisms (NTSR) [10,11]. TSR mechanisms include alterations at the herbicide’s target enzyme, preventing herbicide–enzyme binding or conferring its overexpression and increase in activity [12,13]. NTSR describes any other mechanism not categorized as TSR, such as differential uptake and translocation, vacuolar sequestration, metabolic resistance by enhanced herbicide degradation, or protection against oxidative damage [14]. Despite the high importance of GR weeds to agriculture, their molecular mechanisms are unknown in any species. The high complexity of NTSR mechanisms represents diverse ways that weeds have evolved to deal with the stresses caused by herbicides. It is probable that many of them are still to be understood. A new NTSR mechanism could include the ability to prevent or reduce the amount of herbicide entering the cell in resistant plants.

Transcriptome analysis (RNA-seq) through next-generation sequencing (NGS) is a powerful method for studying global gene expression in organisms [15,16]. This method has been applied successfully in weed science and provided breakthrough data for molecular studies, such as on mechanisms of herbicide resistance [17,18,19,20,21]. Transcriptome studies produce quantitative and qualitative data [22], and the assembling of a transcriptome can be performed without the need for a reference genome (*de novo* assembly). It represents a huge advantage for studies in non-model plants such as LOLMU, which does not have a reference genome available. Likewise, global differential gene expression analysis allows comparisons between GR and GS biotypes with and without herbicide treatment, paving the way to the identification of the most responsive genes involved in the molecular herbicide-resistance mechanisms.

The comparisons of global patterns of gene expression between GR and GS biotypes have the potential to provide the first step towards the identification of the molecular mechanisms of glyphosate resistance in LOLMU. The identification of molecular mechanisms is critical to understanding weed biology, and consequently, to the development of new management strategies to manage herbicide-resistant weeds. In this way, the present transcriptome study aimed to analyze the global gene expression between GR and GS biotypes in response to glyphosate treatment, in order to identify highly responsive genes in the GR biotype and to provide a list of candidate target genes that might be conferring glyphosate resistance in LOLMU.

## 2. Results

### 2.1. Glyphosate Dose–Response and Shikimic Acid Content

**Figure 1:**
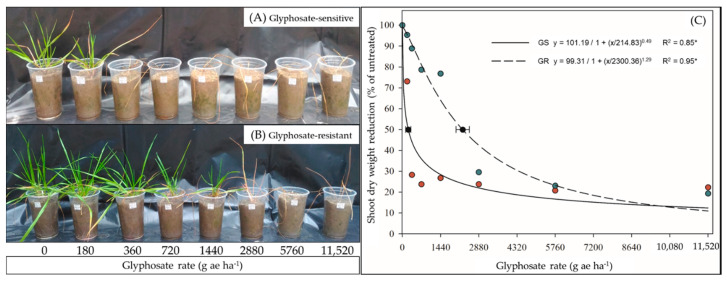
Glyphosate dose–response curves of (**A**,**C**) glyphosate-sensitive (GS) and (**B**,**C**) glyphosate-resistant (GR) biotypes of *Lolium multiflorum* at 28 days after treatment (DAT). (**C**) Lines are the response curves predicted using non-linear regression. Red and green symbols represent mean dry weight (% reduction of untreated) of GS and GR, respectively. The black symbols represent the GR_50_ for each respective biotype and the bars are the confidence intervals (CI: *p* ≤ 0.05).

A glyphosate dose–response experiment was performed to find the glyphosate rate that caused a 50% growth reduction (GR_50_), the resistance factor, and to confirm the resistance/sensitivity status in both GR and GS biotypes. The GR_50_ was 2242 and 248 g ae ha^−1^ for the GR and GS biotypes, respectively, and the resistance factor was 9 (GR_50_ GR/GR_50_ GS). Therefore, these results confirmed that the GR is resistant to glyphosate and that the GS is sensitive to that herbicide (Figure 1).

**Figure 2:**
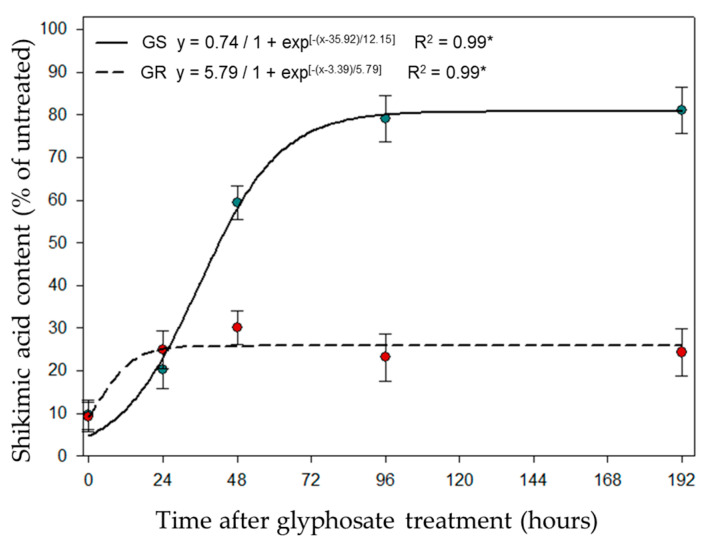
The shikimic acid content of glyphosate-resistant (GR) and glyphosate-sensitive (GS) biotypes of *Lolium multiflorum* in a time-course experiment after treatment with 2160 g ae ha^−1^ of glyphosate.

The results of shikimic acid content (SAC) showed a significant difference between GR and GS. In the GR biotype, there was a slight increase of SAC during the first 24 h, and the levels remained stable after that. In the GS biotype, an accentuated increase of SAC levels occurred from 0 to about 72 h after glyphosate treatment, reaching the highest level at approximately 80 h and stabilizing after that time at high levels (Figure 2).

### 2.2. De Novo Transcriptome Assembly and Functional Annotation

Twelve cDNA libraries from GR and GS biotypes of LOLMU were sequenced on the Illumina platform to compare the global gene expression of glyphosate-treated versus untreated plants. The transcriptome assembling produced 146,263 transcripts, 87,433 contigs at the gene level with an N50 of 740 bp, and the average length of 575.24 bp (Table 1). More than 99.95% of all the raw reads used for *de novo* assembly had a Phred score ≥Q30 level (error probability of *p* ≤ 0.01), demonstrating the high quality of the data. The statistical results showed a decreasing trend of contig number with an increasing length, in which the sequence distribution of transcript length threshold changed from 0 bp to >2500 bp (Figure S1).

The annotation results indicated that 38.8% of the assembled contigs (87,433) were assigned to the nucleotide sequences from the NCBI database using the BLASTx. From the annotated contigs, three species corresponded to about 64% of the mapped genes hits, *Triticum aestivum* (32.68%), *Brachypodium distachyon* (15.86%), and *Hordem vulgare* (15.77%) (Figure S2). The gene ontology (GO) assignments provided the functions of the annotated contigs attributed to at least one GO terms, which were classified into three functional categories: molecular functions, biological processes, and cellular components (Figure S3). The most significant proportion of the annotated specific genes in the biological processes category was attributed to metabolic processes (10%) and protein phosphorylation (9.6%); in the molecular function category, the greatest proportion were attributed to protein kinase activity (9.5%) and oxidoreductase activity (6.4%); and in the cellular components category, an integral component of membrane (4.9%) and nucleus (4.8%) (Figure S3).

### 2.3. RNA-Seq Dataset Validation by qRT-PCR Analysis

**Figure 3:**
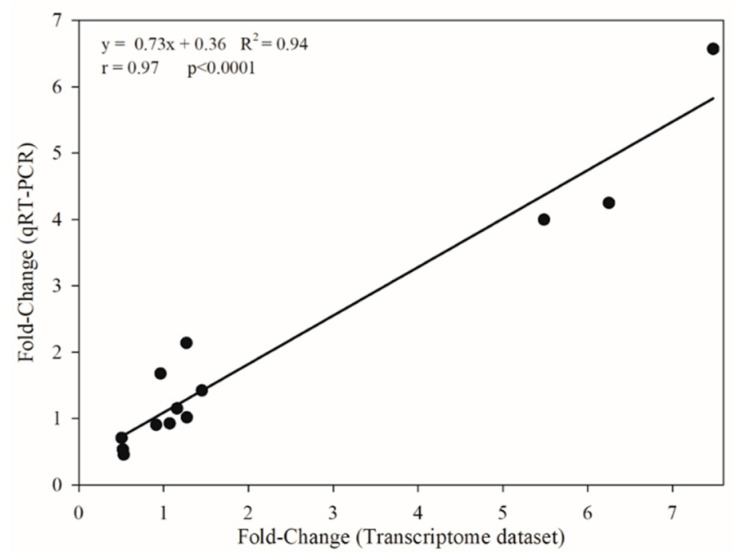
The correlation between transcriptomic (RNA-Seq; X-axis) and qRT-PCR (Y-axis) fold-change (GR/GS) expression levels of 19 genes of glyphosate-resistant (GR) and glyphosate-sensitive (GS) biotypes of *Lolium multiflorum*.

The results of the relative gene expression for 13 genes assessed on qRT-PCR were correlated to those from RNA-seq differential expression analysis. The overall results of gene expression in response to glyphosate treatment presented an amplitude from 0.03 to 275.7 in relative expression in qRT-PCR, whereas for RNA-seq data the amplitude ranged from 0.008 to 78.9 (Figure 3; Table S1). The comparisons between the results obtained from qRT-PCR and RNA-seq indicated a significative correlation of r = 0.97 (*p* < 0.0001), suggesting the reliability of the RNA-seq dataset (Figure 3; Table S1).

### 2.4. EPSPS Sequence Analysis and Expression

The alignment analysis performed by BLASTn identified a single isoform of the EPSPS-coding enzyme sequence in the individual transcriptomes assembled for the GR and GS biotypes. The EPSPS amino-acid sequences from transcriptome aligned significantly with the EPSPS sequence from GenBank (DQ153168.2) with an E-value of zero and scored bits >650. There was no amino-acid substitution at the positions Thr 102 and Pro 106 codons, which are the most commonly reported positions where changes occur in the EPSPS enzyme sequence (Figure S4A). The glyphosate treatment also altered the EPSPS expression levels in the GS biotype, with non-significant differences to the GR biotype (Figure S4B). The fold-change ratio in each biotype in response to glyphosate treatment (t1/t0) indicated that the EPSPS expression increased more significantly in the GS biotype (Figure S4B). Therefore, these results provide evidence that the glyphosate-resistance mechanism in the studied GR *L. multiflorum* biotype is due to NTSR.

### 2.5. Differentially Expressed Genes in Response to Glyphosate

**Figure 4:**
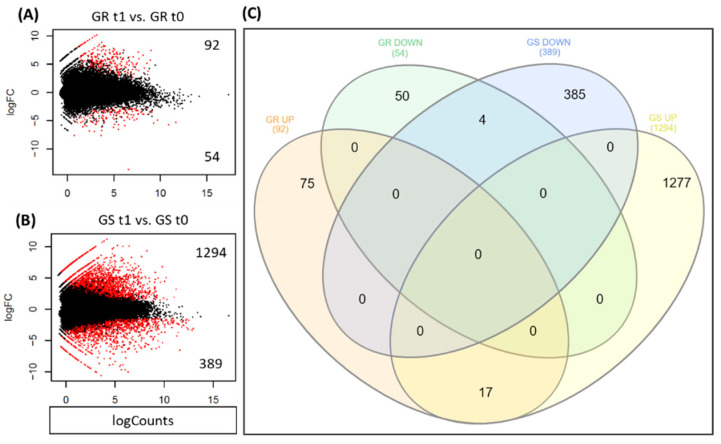
The differential expression analysis from RNA-seq study of *Lolium multiflorum* (**A**) glyphosate-resistant (GR) and (**B**) glyphosate-sensitive (GS) biotypes in response to glyphosate treatment represented in a MA plot (**A**,**B**) and Venn diagram (**C**). Black dots represent non-significant expression, while red dots indicate significant expression. The expression threshold was a *p*-value and false discovery rate (FDR) of ≤0.05, and log2 FC ≥2 (up-regulated) or ≤log2 FC (down-regulated). In the MA plots, the X-axis (M) corresponds to the mean of normalized counts and the Y-axis (A) to the log2 fold-change for each contig. t1 means with glyphosate treatment and t0 without treatment. Two biotypes originating from the same geographical region (São Valentin, RS, Brazil) were used, one GR and the other GS.

The differentially expressed genes (DEGs) in response to glyphosate treatment for the GR and GS biotypes were summarized in a M (log ratio) and A (mean average) scales (MA plot) and a Venn diagram, showing the gene expression patterns as well as the overlapping relationship using threshold cut-off log2 FC ratio (≥2 up-regulated and ≤−2 down-regulated), *p*-value, and false discovery rate (FDR) set at ≤0.05. A total of 1829 genes were differentially expressed in both GR and GS biotypes, with 1386 (75.8%) being up- and 443 (24.2%) being downregulated (Figure 4A,B). From the total DEGs, 146 were observed in the GR biotype and 1683 in the GS. In response to glyphosate treatment, the GR biotype presented 92 (6.6%) upregulated and 54 (12.2%) downregulated genes, whereas the GS had 1294 (93.4%) up- and 389 (87.8%) downregulated, respectively (Figure 4). Among all DEGs, 21 were differentially expressed in both GR and GS biotypes in response to glyphosate treatment. Of those, 17 were upregulated and 4 downregulated (Figure 4C). From those 21 DEGs, five upregulated (UDP-glucosyltransferase UGT13248, Sulfate transporter, Glucan endo-1,3-beta-glucosidase GLC1, COBRA-like protein 7 COBL7, and Ubiquitin) and one downregulated gene (Vacuolar protein sorting-associated - VPS60-1) met the selection criteria (log2 FC ≥2, ≤−2, FDR, and *p*-value of ≤0.05) and are included among the candidate gene target list provided in this study (Table 2).

**Figure 5:**
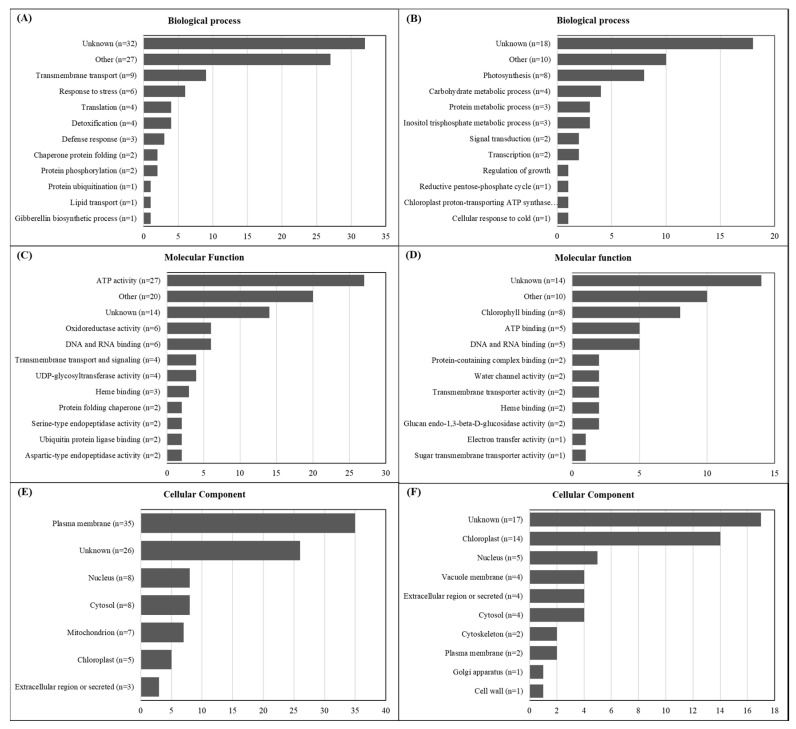
Summary of top twelve gene ontology (GO) terms identified as up- (**A**,**C**,**E**, n = 92) and downregulated (**B**,**D**,**F**, n = 54) genes (DEGs) in the glyphosate-resistant (GR) biotype of *L. multiflorum* transcriptome in response to glyphosate treatment. The sequences were annotated and further classified into biological processes, molecular functions, and cellular components. Two biotypes originating from the same geographical region (São Valentin, RS, Brazil) were used, one GR and the other GS.

The gene ontology analysis performed for the 146 DEGs (92 up- and 54 downregulated) from the GR biotype in response to glyphosate treatment indicated that on average, 28.1% of the genes were from unknown processes from all three categories (biological processes, molecular functions, and cellular components) (Figure 5). For upregulated characterized genes, transmembrane transport (n = 9, 9.8%) and response to stress (n = 6, 6.5%) presented the highest number in biological processes; ATP activity (n = 27, 29.3%) and oxidoreductase activity (n = 6, 6.5%) in molecular functions; and plasma membrane (n = 35, 38%) and nucleus/cytosol (n = 8, 8.7% each) were the most representative gene ontologies in cellular components (Figure 5A,C,E). The same comparisons for downregulated genes showed that photosynthesis (n = 8, 14.8%) and carbohydrate metabolic process (n = 4, 7.4%) had the highest number of genes in biological processes; chlorophyll-binding (n=8, 14.8%) and ATP binding (n = 5, 9.3%) in molecular functions; and chloroplast (n = 14, 25.9%) and nucleus (n = 5, 9.3%) in cellular components (Figure 5B,D,F).

### 2.6. Glyphosate Resistance Candidate Gene List

A total of 21 DEGs were selected as candidates to be involved in glyphosate resistance mechanisms in LOLMU. From those, 14 were up-regulated and 7 down-regulated genes (Table 2). Among the up-regulated genes, there were genes known to be involved in herbicide-resistance mechanisms in weeds, such as three glycosyltransferases and one ABC transporter. These genes have been reported in several studies to be related to the conjugation of herbicides or their subproducts after degradation to sugars and other molecules, and their transport into vacuoles or extracellular compartments, respectively [23,24]. In addition, our report presents ten new upregulated genes that were highly responsive to glyphosate treatment in the GR biotype (Table 2). The functions of the new genes were related to cell wall integrity and stress responses, cellulose organization (COBRA-like protein), oxidoreductase activity (Fe2OG dioxygenase), gibberellin oxidase, defense response (glucan endo-1,3-beta-glucosidase), response to wounding (subtilisin-chymotrypsin inhibitor), sulfate transporter, protein degradation (ubiquitin), and an uncharacterized protein which is a component of the membrane and presented the highest level of response in GR biotype (Table 2). On the other hand, the seven down-regulated genes were related to the oxidoreductase process (DAO-domain protein), cell-wall organization (glycine-rich cell wall structural protein), RNA polymerase, senescence-associated protein, transcription factor, an uncharacterized protein with no characterized cellular compartment, and vacuolar protein-sorting-associated (Table 2).

Among the candidate gene list, there were three differentially expressed genes in the GR biotype that were not detected in the GS biotype. The ubiquitin-60S ribosomal protein was upregulated (log2 FC 6.4) whereas the senescence-associated protein and uncharacterized protein (gene TRAES_3BF019300240CFD_c1) were downregulated (log2 FC −5.2 and −5.4, respectively) (Table 2). We also highlighted the up-regulated genes in the GR biotype in response to glyphosate treatment that presented very low expression in the GS biotype. These genes were related to gibberellin 20 oxidase (log2 FC in GR 4.9 vs. 0.6 in GS) and the uncharacterized protein component of membrane (log2 FC in GR 4.9 vs. 0.5 in GS) (Table 2). These comparisons resulted in the highest fold-change when comparing the gene expression in the two biotypes (GR/GS), which was 8.7 and 9.9, respectively (Table 2). The very interesting result of the all up- and down-regulated genes in the list is that a great proportion of them were related to membrane processes or located at the cell wall, plasma membrane, integral components of the membrane, and extracellular regions. Genes involved in nuclear or cytoplasmic process also had a relevant proportion among all DEGs (Table 2).

## 3. Discussion

The present study is the first transcriptome analysis reported for the *L. multiflorum* species, and providing a large amount of high-quality data. The molecular data provided here will support future general molecular studies in this species, as well as serving as a dataset for further studies towards understanding the glyphosate-resistance mechanisms in LOLMU. The 87,433 annotated contigs in the LOLMU transcriptome contained the most responsive molecular changes in the GR and GS biotypes in response to glyphosate treatment. The genes that will lead us to the mechanisms of resistance to glyphosate in LOLMU are most likely represented among the differentially expressed contigs.

The high proportion of non-annotated contigs’ functions demonstrate the necessity for the characterization of weed genomes—in this particular case, for *L. multiflorum* or other closely related species. The characterization of the LOLMU transcriptome will support molecular studies in the weed science field, such as weediness and the evolution of herbicide-resistance mechanisms [25]. Since there is no available LOLMU genome and knowing that weed genomics is essential to the study of weed biology, and understanding of weed biology is critical for weed management [25], the present LOLMU transcriptome will serve as a useful dataset for this type of study. The differential gene expression in both GR and GS biotypes in response to glyphosate treatment will also help to identify candidate genes and mechanisms involved in the evolution of glyphosate resistance.

The non-occurrence of nucleotide substitution at Thr 102 and Pro 106, the absence of a significant increase in EPSPS gene expression in GR biotype, and the presence of a single coding-gene sequence in both GR and GS biotypes provided evidence that the glyphosate resistance in the studied GR LOLMU biotype is due to a non-target-site resistance (NTSR) mechanism. Another study using the same GR and GS LOLMU biotypes investigated alterations in EPSPS coding sequences through Sanger sequencing and also concluded that there were no alterations at the 102 and 106 codons [26]. We also verified the EPSPS expression by qRT-PCR, and both methodologies, RNA-seq and qRT-PCR, indicated relatively higher responses in GS than in GR in response to glyphosate treatment (Table S1; Figure S4).

The gene ontology assignments performed for the up- and down-regulated genes in GR as a response to glyphosate treatment demonstrated a significant proportion of up-regulated genes classified into the transmembrane transport (Figure 5). These genes also represented the highest proportion of those annotated in the cellular component. On the other hand, genes related to photosynthesis processes into the chloroplasts were the most abundant down-regulated genes (Figure 5). The great number of highly glyphosate-induced genes related to cell membrane processes in the GR biotype indicated that the responses to glyphosate action stimulate the plant defenses to create a barrier to reduce the amount of or prevent entirely the glyphosate entering the cell. Plant cells are surrounded by highly dynamic cell walls and plasma membrane, performing significant roles in plant development [27]. The cell wall and membrane are the first layer of protection against abiotic stresses [27], which might include herbicide [28]. The detection of potential stressors to the cell by receptors triggers several coordinated signaling events that result in the production of protective metabolites, cell-wall and membrane remodeling, or even cell death [27,28].

Glyphosate directly inhibits the enzyme EPSPS, which results in an interruption of the shikimic acid pathway and consequent disruption of aromatic amino acid biosynthesis [29]. Interruption of the shikimic acid pathway will trigger an accumulation of shikimic acid [30]. A previous study in *Conyza canadensis* reported a linear correlation of shikimic acid content with the amount of glyphosate transported into the cells [31]. In the present study, the low accumulation of shikimic acid in the GR biotype is strong evidence of the reduced amount of glyphosate entering the cells and reaching its target, the EPSPS enzyme (Figure 2). Hence, another study with the same biotypes evaluated high rates of glyphosate (0–11,520 g ae ha^−1^) found that increasing the glyphosate rate did not increase the shikimic acid accumulation at 48 h after treatment [26]. Additionally, the significant number of down-regulated genes involved in the photosynthesis process in the GR biotype indicated the occurrence of signaling for plant metabolism slowdown as a coping mechanism against glyphosate action. This will ameliorate the plant’s defenses against the glyphosate movement and damage. The reduction in plant metabolism is the initial response to abiotic stress [32].

The reduced number of up- and down-regulated genes in the GR biotype (146) indicated that glyphosate treatment did not induce significant molecular changes when compared to the GS biotype, which had over a thousand DEGs (1683) (Figure 4). The low number of DEGs in the GR biotype is further evidence of the reduced amount of glyphosate entering the cell, because glyphosate action causes great disturbances in plant physiology and metabolic processes [33]. Because of this, it was expected that a great number of DEGs would be observed for the GR biotype. Recent transcriptome studies seeking glyphosate-resistance mechanisms in *Conyza bonariensis* [17,18] and *Echinochloa colona* [34] found about 4500 DEGs in response to glyphosate in GR biotypes and similar results in GS. After glyphosate reaches and binds to EPSPS, it interrupts the shikimic acid pathway and the biosynthesis of aromatic amino acids (phenylalanine, tyrosine, and tryptophan) [30]. The inhibition of the shikimic acid pathway leads to an accumulation of shikimic acid, reducing power (NADPH+H), production of reactive oxygen species (ROS), lipidic peroxidation, and membrane disintegration, ultimately leading to cell death [30,33]. Therefore, glyphosate action results in wide perturbation of the plant’s metabolic system [35]. In the present study, the alterations on gene expression in the GR biotype were low. Still, the results enabled the selection of a narrow and effective candidate gene list for involvement in glyphosate resistance mechanisms.

Among the differentially expressed genes from the RNA-Seq analysis, we selected the 21 most responsive to glyphosate treatment in GR in comparison to GS, 14 being up-regulated and 7 down-regulated (Table 2). In the up-regulated candidate gene list, two groups of well-known genes established to be involved in herbicide conjugation and transport were the most induced by glyphosate, glycosyltransferase (GTs) and ABC transporters [23,24]. In general, it is well accepted that herbicide NTSR, usually metabolism and degradation, follows a four-phase process to protect the plant against irreversible herbicide damage and death: first—oxidation, second—conjugation, third—transport, and fourth—degradation, detoxification, and protection [10,11,24,36,37]. On the candidate target gene list reported in the present study, there are three upregulated GTs and one ABC transporter. GTs are in the cytoplasm and conjugate lipophilic molecules, such as herbicides, directly or to several substrates, which results in a polar product favoring its transport or pumping into vacuoles by ABC transporters [10,14,37,38].

The candidate gene list also includes additional highly induced genes that most likely act to prevent the glyphosate molecule of transposing through the cell wall and plasmatic membrane (Table 2). From the 14 upregulated genes, nine are related somehow to membrane processes. Once this type of mechanism is proven, the prevention of herbicide entering the cell might be a new phase added to the four phases of NTSR mechanisms. Recently, an article reported that the aldo-keto reductase metabolizes glyphosate and confers resistance in *E. colona* [34]. We investigated the transcription levels of the aldo-keto reductase in the LOLMU transcriptome and found no difference in expression between GR and GS biotypes with or without glyphosate treatment.

Two up-regulated genes were annotated as ubiquitin (Table 2), which is a type of protein that exists freely or conjugated to another protein. In general, ubiquitin is involved in protein posttranslational modification or degradation via the proteasome. However, it is also involved with the activation of the protein kinases and cell signaling [39,40]. Proteins and regulators from several processes are targeted by ubiquitin for further degradation at the proteasome, allowing the cells to maintain cellular responses to environmental changes, such as abiotic stresses [39]. The role of ubiquitin during abiotic stress, such as herbicide exposure, involves controlling the protein load in the cell, which will affect many cellular activities, including signaling and gene expression [39]. As glyphosate inhibits the biosynthesis of amino acids, a possible hypothesis that still needs to be determined for increasing ubiquitin expression is that it works to increase protease activities in order to release free amino acids upon herbicide treatment [41]. Another hypothesis might be the involvement of ubiquitination on the degradation of toxic proteins produced after glyphosate action.

In addition to GTs, ABC transporters, and those genes related with activities on the cell membrane, assuming that their functions prevent glyphosate entering into the cell, the participation and function of the other glyphosate-resistance candidate genes reported in Table 2 on molecular responses to glyphosate action are yet to be determined (up-regulated genes related to oxidoreductase activity (Fe2OG dioxygenase), gibberellin oxidase; and down-regulated genes related to oxidoreductase process (DAO-domain protein), cell-wall organization (glycine-rich cell wall structural protein), RNA polymerase, senescence-associated protein, transcription factor, uncharacterized protein, and vacuolar protein-sorting-associated).

In the present study, two biotypes were studied. Future studies should increase the number of populations of LOLMU to clarify the representativeness of this mechanism in the field. Further genomic data and complete characterization of the gene ontology, as well as functional genomics of the *L. multiflorum* or related species, will be helpful to validate the mechanisms of glyphosate resistance suggested by the target gene list. In the meantime, the present data will drive further studies on functional genomics of this narrow group of 21 target genes towards underlying the mechanisms of glyphosate resistance. Techniques such as genome editing approaches, e.g., CRISPR/Cas9 systems, could be used to knock out the candidate genes for further phenotyping evaluations and validation of the mechanism. Labeled-glyphosate studies could also be performed to evaluate glyphosate movement at the plant level, as well as at the cellular level using protoplasts (cells without a cell wall) and cell culture approaches (cell with cell wall).

## 4. Material and Methods

### 4.1. Glyphosate Dose–Response and Whole-Plant Shikimic-Acid Bioassay

Two biotypes of LOLMU, GR (SVA04) and GS (SVA02), originating from São Valentin-RS/Brazil (27.35° S, 54.28° W) were vegetatively multiplied (separating tillers), transplanted to individual pots, and grown in a greenhouse. Glyphosate dose–response experiments were performed, following the official criteria to determine the dose required to cause 50% of growth reduction (GR_50_) in both GR and GS biotypes [42]. The Roundup Original 360 SL (Monsanto - Brazil) was applied on 4–6-leaf-stage plants at 0, 180, 360, 720, 1440, 2880, 5660, and 11,520 g ae ha^−1^ using a CO_2_ backpack sprayer delivering 120 L ha^−1^.

The quantification of the shikimic acid content (SAC) was performed according to Singh and Shaner and Perez-Jones et al. [43,44] with previously described modifications [45]. The top three leaves of GR and GS biotype plants were harvested after glyphosate treatment and immediately stored at −80 °C. The time-points used for SAC determination were 0, 24, 48, 96, and 192 h after treatment with 2160 g ae ha^−1^ of glyphosate. Fresh weight samples of 0.25 g were harvested from leaves, chopped and homogenized in 5 mL of 1.25 N HCl solution, and frozen at −80 °C. Samples were kept at room temperature (22 °C) for approximately 15 min, then incubated at 37 °C for 45 min. Subsequently, 125 µL per technical sample (total of five technical samples) was collected and mixed with the reaction buffer (0.25% (*w/v*) periodic acid and sodium(meta)periodate solution) and incubated at 37 °C for 30 min. This reaction allowed the oxidation of the shikimic acid. After incubation, an aliquot of 1000 µL of 0.6 N NaOH/0.22 M Na_2_SO_3_ was added to the sample. After that, the shikimic acid was measured spectrophotometrically at 380 nm using a cuvette, and the SAC was determined using a standard curve in µg.g^−1^ fresh weight (µg.g^−1^ FW). The results were expressed as percentage of SAC in relation to the control.

### 4.2. RNA-Seq Experimental Design and RNA Extraction

**Figure 6:**
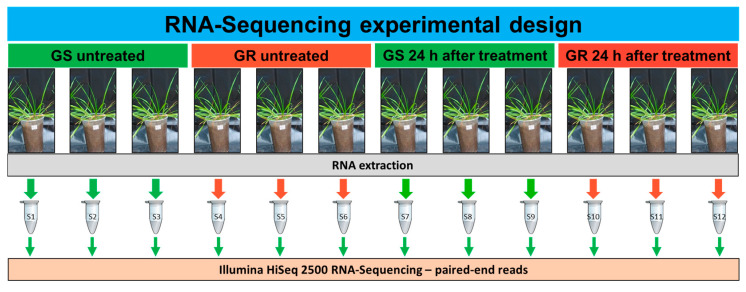
The RNA-seq experimental design using glyphosate-resistant (GR) and glyphosate-sensitive (GS) biotypes of *Lolium multiflorum*. RNA samples 1–3 and 4–6: GS and GR untreated, respectively; RNA samples 7–9 and 10–12: GS and GR, respectively, treated with glyphosate at 2160 g ae ha^−1^ and harvested at 24 h after treatment. Three biological replicates were used per treatment (GS t0, GR t0, GS t1, and GR t1—a total of 12 libraries). Two biotypes originating from the same geographical region (São Valentin, RS, Brazil) were used, one GR and the other GS.

The RNA-seq experimental design included six biological replicates each of the GR and GS biotypes, three with glyphosate treatment (2160 g ae. ha^−1^) and three without treatment, giving a total of 12 plants. Three plants of each GR and GS biotypes were treated with glyphosate at 2160 g ae ha^−1^, according to the method described above. At 24 h after treatment, the second and third leaves (from the apex) from all treated and non-treated plants were harvested and immediately frozen in liquid nitrogen and stored at −80 °C. Each plant formed an individual sample (Figure 6).

The Trizol reagent (Invitrogen, Carlsbad, Calif, USA) was used for the RNA extraction following the company’s recommendation. The residual genomic DNA was removed with DNase I (Invitrogen). The final experimental design comprised 12 RNA samples: 3 GS untreated (GS t0), 3 GR untreated (GR t0), 3 GS treated (GS t1), and 3 GR treated (GR t1) (Figure 6).

### 4.3. cDNA Library Construction and Illumina Sequencing

The cDNA library preparation and Illumina sequencing were performed at the Laboratory of Functional Genomics Applied to Agriculture and Agri-Energy, University of São Paulo (USP), Piracicaba, Brazil. The RIN (RNA integrity number) values and concentration of each sample were examined in the Agilent 2100 Bioanalyzer (Agilent Technologies, USA). A reference cDNA library was constructed using 500 ng of total RNA samples and, the mRNA was enriched and purified according to the Illumina TruSeq Stranded mRNA Sample Preparation Kit (Illumina, San Diego, CA) following the manufacturer’s LT protocol to break it into short fragments with incubating mix for 8 min at 94 °C. The first-strand cDNA was synthesized by adding the Superscript II reverse transcriptase (Invitrogen), followed by thermal cycle incubation at 25 °C for 10 min, 42 °C for 15 min, and 70 °C for 15 min. The cDNA plate (CDP) barcode was removed, and the second-strand synthesis proceeded after addition of the master mix at 16 °C for 60 min. Subsequently, end-repair was performed to remove the 3′ overhangs and proceed immediately to ligate adapters at the 5′ and 3′ ends of each strand in the DNA fragment, which was important for library amplification during cluster formation. The DNA fragments were enriched in a preheated thermal cycler using 1 cycle at 98 °C for 30 s, 15 cycles at 98 °C for 10 s, 60 °C for 30 s, and 72 °C for 30 s, followed by one cycle at 72 °C for 5 min. Finally, the 12 libraries were sequenced using the HiSeq Flow Cell v4, with the Illumina HiSeq 2500, producing 125 bp paired-end reads (2×).

### 4.4. De Novo Transcriptome Assembly and Functional Annotation

Preprocessing was performed using the raw data to remove low-quality reads (ambiguous sequence ‘N’ or very short sequences), adaptors, and contamination. The preprocessing was performed with the Fastqscreen tool [46], followed by the checking of sequence quality using the FastQC (https://www.bioinformatics.babraham.ac.uk/). All raw reads were submitted to the preprocessing to trimming and quality filtering (*option*: LEADING:3, TRAILING:3, SLIDING WINDOW:4:15), and to remove the adapter sequences (*option*: ILLUMINACLIP 2:40:15) using the Trimmomatic [47] which were used to reconstruct a full-length transcriptome.

The clean reads were assembled *de novo* using Trinity [48,49] with the default settings except for the K-mer value (25 mer), and transcripts under 300 bp discarded during assembly. The transcriptome assembly, used for differential gene expression, was performed for all 12 libraries (all samples and treatments), producing a single *de novo* transcriptome, which is recommended as an essential step for differential expression analysis [48,49,50]. For EPSPS sequences analysis, a single *de novo* transcriptome was assembled for both GR and GS biotypes. Trinity was executed using the default settings, and at the end of the assembly, statistics (e.g., N50, L50, CG%) were calculated using the accessory script “*TrinityStats.pl*”. The BioPython package [51] was used to do additional assembly analysis. After assembling, the transcripts were aligned to the UniProt-trEMBL [52] database using Diamond [53].

Annotation was performed using the Trinotate pipeline, using the Pfam [50] UniProt-SwissProt database to identify the protein families, and SignalP [54] and TMHMM [55] to identify transmembrane proteins and peptides, respectively. The prediction of rRNA transcripts was performed using the RNAmmer [56]. A BLAST with a significance threshold of E-value ˂10^−5^ was used to compare all assembled unigenes with the non-redundant proteins from Swiss-Prot, TrEMBL, CDD, Pfam, and KOG databases.

Gene ontology (GO) terms were used to evaluate the functional categories of the best BLASTX hits from the non-redundant protein database with the BLAST2GO software with an E-value threshold of 10^−5^, grouping by molecular function, biological process, and cellular component. The unigenes were subjected to clusters of orthologous groups for eukaryotic complete genomes (KOG) classification to evaluate the integrity of the transcriptome library and the effectiveness of the annotation process.

### 4.5. Differential Gene Expression Analysis and Candidate Target Selection

In the differential expression analysis, we assumed and termed each contig as a “gene.” The RNA-Seq data were normalized, and the gene expression determined through transcripts reads per million mapped reads (TPM ≥ 2). For each replicate, an estimation of gene expression was made using the Kallisto method [57] implemented in the Trinity accessory, which generated the expression matrix. The differential gene expression was assessed in the edgeR mode by processing the expression matrix, GO binning, and enrichment [58].

Differentially expressed genes (DEGs) were contrasted within each biotype (t1 vs. t0) using the false discovery rate (FDR) and *p*-value threshold set of ≤0.05, and then the lists of all DEGs were exported for each comparison. MA plots were produced within DESeq2, and the DEG lists were filtered to remove genes with log2 fold-change values (log2 FC) less than 2 and higher than −2. In this case, DEGs with a log2 fold-change ≥2 (log2 FC ≥2) were considered up-regulated, while ≤−2 (log2 FC ≤−2) were considered down-regulated. A Venn diagram was produced using all DEGs from the GR and GS biotypes in response to glyphosate treatment [59]. The up- and down-regulated genes for GR biotype were categorized according to GO functions using the methods described above.

### 4.6. RNA-Seq Dataset Validation through qRT-PCR

The same RNA samples as were used for RNA-sequencing were used for quantitative reverse transcriptase polymerase chain reaction (qRT-PCR) analysis. The RNA integrity was assessed in agarose gel electrophoresis at 1% (p/v), while the concentration and purity were measured in a NanoDrop^TM^ 2000 spectrophotometer (Thermo Scientific). The RNA was converted to cDNA using the SuperScript^TM^ First-Strand Synthesis System Kit according to the manufacturer’s methodology.

The qRT-PCR analysis was carried out in a Light Cycler 480 Instrument II (96)^TM^ (Roche Applied Science^TM^), using three biological replicates of cDNA. Amplification was performed with 6.25 μL of SYBR Green I Master (Roche Applied Science), 0.5 μM of primers (10 mM), 1 μL of cDNA (0.2 μg), and 4.25 μL ultrapure water, giving a final volume of 12 μL. The qRT-PCR parameters were denaturation cycle at 95 °C for 5 min, followed by 45 cycles at 95 °C for 20 s, 60 °C for 15 s, and 72 °C for 20 s, which was followed by a dissociation curve with denaturation at 95 °C for 5 s, cooling at 70 °C for 1 min, and gradual warming at 0.11 °C to 95 °C and cooling at 40 °C for 30 s. The amplification was verified by the presence of a single peak on the qRT-PCR melting curve and a single band with the expected size in the 2% of agarose gel electrophoresis.

We evaluated the stability of four candidate reference genes previously reported as 18s ribosomal protein (18s) [60], glyceraldehyde 3-phosphate dehydrogenase (GAPDH) [61], alpha-tubulin 5 (TUA5) [62], and eukaryotic elongation factor 1 alpha (eEF1As) [63] (Table S2). The stability of the expression of candidate reference genes was evaluated via qRT-PCR for all cDNA treatments (GR and GS with and without application of glyphosate). The cycle threshold (Ct) values were analyzed by RefFinder software [64], indicating that the 18s and eEF1As were the most stable reference genes across the treatments. Therefore, the average of the results for both 18s and eEF1As was used to normalize the qRT-PCR data. The relative expression was calculated using the delta–delta ct method RQ = 2^−(∆∆CT)^ [65]. A total of 13 genes from different levels of expression were randomly selected from the general DEG list to get a range of expression, and qRT-PCR was performed. The primers for the 13 evaluated genes are shown in Table S3. The Pearson model (r) was used to correlate the qRT-PCR and RNA-seq results. The results of expression between GR and GS biotypes were compared by F-test at *p* ≤ 0.05 [65].

### 4.7. EPSPS Transcript Sequence Analysis

The sequence of the single-copy EPSPS gene of *L. multiflorum* was obtained from GenBank (DQ153168.2) and mapped into the individual transcriptomes from the GR and GS biotypes to identify their respective contigs. The mapping was performed using BLASTn command-line. Additionally, the EPSPS sequences of *Arabidopsis thaliana* (GenBank CAA29828.1) and *Zea mays* (GenBank AF349754) were used for comparisons. Trinity-assembled EPSPS sequences for LOLMU GR and GS biotypes, and those from *L. multiflorum, A. thaliana*, and *Z. mays* from GenBank were converted to amino acids. Next, all sequences were aligned into BioEdit (http://www.mbio.ncsu.edu/BioEdit/) using the ClustalW multiple alignment functions with default settings. The amino acid substitutions at threonine 102 and proline 106 were evaluated because these are commons coding regions conferring resistance to glyphosate in weeds [12,17]. The transcription level of the EPSPS contigs was also analyzed to verify the changes in expression in both GR and GS after glyphosate treatment, as well as the number of copies.

### 4.8. Selection of Differentially Expressed Candidate Genes

The DEGs were selected with a focus on the GR biotype responses to glyphosate treatment after comparisons with the GS biotype. The expression levels of genes with log2 FC ≥2, ≤−2, FDR, and *p-*values of ≤0.05 were selected for the GR biotype in response to glyphosate treatment, i.e., GR t1 vs. t0. The log2 FC results from all selected DEGs from GR were contrasted with those from GS ((GRt1/GRt0)/(GSt1/GSt0)). DEGs that presented a significant difference by F-test (*p* ≤ 0.05) between GR and GS biotypes were selected.

## 5. Conclusions

The present study is the first report of transcriptomic data for *Lolium multiflorum,* which will provide a large quantity of high-quality data for further molecular investigations in this species, since there is no genome available. Glyphosate treatment did not induce high molecular responses in the glyphosate-resistant biotype, while it did so in the glyphosate-sensitive biotype. The gene ontology analysis showed that most of the up-regulated genes in the resistant biotype were associated with response to stress. In contrast, the down-regulated genes were mostly associated with photosynthesis, indicating a slowing down of plant metabolism. We also reported a list with 21 candidate genes that may be involved with the non-target-site mechanisms of glyphosate resistance in *Lolium multiflorum.* Among the candidate gene list, glycosyltransferases and ABC transporters have well-known involvement in the herbicide resistance process. The gene list also contains nine up-regulated genes that are involved in the plasma membrane, and other up- and down-regulated genes with functions that need to be determined in relation to herbicide metabolism. The high proportion of up-regulated genes involved in the membrane process and the low levels of shikimic acid accumulation in the glyphosate-resistant biotype after treatment with glyphosate provide evidence for reduced amounts of herbicide entering the cell and reaching its target site, the EPSPS enzyme. The present study will serve as an initial step towards understanding the mechanisms of glyphosate resistance in *Lolium multiflorum*. Future work will involve functional genomic and genome-editing approaches to validate the candidate genes’ participation in the resistance process.

## Figures and Tables

**Table 1:** Summary of the Illumina sequencing and de novo transcriptome assembly statistics of glyphosate-resistant and -sensitive biotypes of *Lolium multiflorum*.

Description	Assembly Statistics ^*^
Total assembled bases	155,613,732
Total transcripts	146,263
Total contigs at “gene” level	87,433
Contig N50	740
Average contig length (bp)	575.24
GC (%)	50.47
Phred score ≥Q30	99.95%

* Assembled from all biotypes and treatments, a total of 12 libraries. Two biotypes originating from the same geographical region (São Valentin, RS, Brazil) were used, one GR and the other GS.

**Table 2:** The candidate gene list for involvement in glyphosate-resistance mechanisms in *Lolium multiflorum* obtained from differential expression analysis from RNA-seq in glyphosate-resistant (GR) and glyphosate-sensitive (GS) biotypes in response to glyphosate treatment. The fold-change ratio represents the ratio of the gene expression between GR/GS biotypes in response to glyphosate treatment ((GRt1/GRt0)/(GSt1/GSt0)).

Contig ID	Uniprot ID	Functional Annotation—Gene	Putative Localization	^1^ Log2 FC GR t1/t0	^1^ Log2 FC GS t1/t0	Fold-Change GR/GS *
**Upregulated Genes**						
DN140736_c3_g2	Q9STT5	*ABC transporter A family member 7-ABCA7*	Multi-pass membrane protein	4.8	1.9	2.5
DN134601_c1_g3	P53832	*Cell wall integrity and stress response-WSC2*	Plasma membrane	6.6	4.5	1.4
DN140896_c3_g3	Q8GZ17	*COBRA-like protein 7-COBL7*	Cell membrane	4.6	2.8	1.7
DN138383_c1_g1	A0A3B6IQ15	*Fe2OG dioxygenase domain-containing protein*	Unknown	4.0	2.4	1.7
DN112601_c0_g2	Q39111	*Gibberellin 20 oxidase 2-GA20OX2*	Unknown	4.9	0.6	8.7
DN137545_c0_g2	P52409	*Glucan endo-1,3-beta-glucosidase-GLC1*	Component of plasma membrane	3.7	2.3	1.6
DN130084_c1_g4	M0Y4P1	*Glycosyltransferase UDP UGT13248*	Intracellular-membrane-bound	3.5	2.7	1.3
DN125138_c7_g3	Q7XT97	*Glycosyltransferase UDP 79 UGT79*	Intracellular-membrane-bound	3.8	2.9	1.3
DN123097_c0_g1	A0A3B6PNF9	*Glycosyltransferase*	Unknown	4.8	2.7	1.8
DN111587_c0_g1	P82977	*Subtilisin-chymotrypsin inhibitor-WSCI*	Extracellular region or secreted	5.9	3.7	1.6
DN135754_c1_g1	Q9MAX3	*Sulfate transporter 1.2*	Multi-pass membrane protein	3.6	2.1	1.7
DN120520_c4_g4	P69326	*Ubiquitin*	Nucleus and cytoplasm	6.3	3.4	1.8
DN123915_c1_g2	P0CH35	*Ubiquitin-60S ribosomal protein L40-2 Ub-CEP52-2*	Nucleus and cytoplasm	6.4	N/A	6.4
DN141149_c4_g5	A0A3B6THG7	*Uncharacterized protein*	Integral component of membrane	4.9	0.5	9.9
**Downregulated Genes**						
DN134059_c0_g2	A0A3B6MSN4	*DAO domain-containing protein*	Unknown	−3.1	−1.2	2.5
DN119890_c0_g2	P17816	*Glycine-rich cell wall structural protein-GRP*	Cell wall	−4.5	−2.0	2.2
DN137034_c0_g1	A0A3B5Y7P6	*RNA polymerase sigma factor*	Chloroplast	−2.9	−1.3	2.2
DN136212_c1_g1	P27626	*Senescence-associated protein-DIN1*	Unknown	−5.2	N/A	5.2
DN134440_c0_g3	Q41558	*Transcription factor-HBP-1b(c1)*	Nucleus	−3.5	−1.5	2.4
DN124989_c0_g4	W5D4P6	*Uncharacterized protein-TRAES_3BF019300240CFD_c1*	Unknown	−5.4	N/A	5.4
DN127114_c0_g3	Q9LPN5	*Vacuolar protein sorting-associated-VPS60-1*	Endosome	−4.1	−2.1	2.0

^1^ Positive numbers indicate upregulated genes, whereas negative numbers indicate downregulated genes in response to glyphosate treatment using the log2 FC criteria (numbers <1 and >0 will be negative). * The fold-change results were significant by the F test (*p* ≤ 0.05). The results of differential expression were filtered using the thresholds of false discovery rate (FDR) and *p*-value set at ≤0.05, as well as log2 FC of ≥2 for upregulated and ≤−2 for downregulated genes. N/A indicates gene expression not detected. t0 = without glyphosate treatment; t1 = with glyphosate treatment at 24 h after treatment. Two biotypes originating from the same geographical region (São Valentin, RS, Brazil) were used, one GR and the other GS.

## Data Availability

The RNA-Seq data generated in this study have been uploaded into the NCBI-SRA database under bio project PRJNA521415 ID: 521415 and the accession numbers SRA: SRX5765032- SRX5765043. Available at: https://www.ncbi.nlm.nih.gov/bioproject/PRJNA521415/.

## Supplementary Materials

Supplementary materials can be found at https://www.mdpi.com/2223-7747/9/6/685/s1. Figure S1. Length distribution of transcripts assembled from RNA-Seq libraries of glyphosate-resistant and -sensitive biotypes of *Lolium multiflorum,* Figure S2. Sequence comparison to other plants (hit ≥ 1%) from the distribution of BLASTx hits (e-value < 1e−10) against the non-redundant protein database of the National Center for Biotechnology Information (NCBI), Figure S3. Top twelve Gene Ontology (GO) terms identified in the *Lolium multiflorum* transcriptome assembly summarized in three main categories, Figure S4. Partial sequence alignment of the EPSPS transcripts and amino acid sequence assembled of transcriptomes from glyphosate-resistant (GR) and -sensitive (GS) biotypes compared to ^1^EPSPS sequence of the *Arabidopsis thaliana* (GenBank: CAA29828.1); ^2^EPSPS sequence of *Lolium multiflorum* (GenBank: DQ153168.2); ^3^EPSPS sequence of *Zea mays* (GenBank: AF349754), Table S1. The gene expression results of qRT-PCR (relative gene expression) and RNA-Seq (fold-change) in response to glyphosate treatment in glyphosate-resistant (GR) and -sensitive (GS) biotypes of *Lolium multiflorum* used to validate the transcriptome dataset, Table S2. Candidate reference genes for normalization of qRT-PCR in *Lolium multiflorum* glyphosate-resistant and -sensitive biotypes in response to glyphosate treatment, Table S3. The primers for 13 genes used for qRT-PCR analysis in *Lolium multiflorum* glyphosate-resistant and -sensitive biotypes in response to glyphosate treatment.
