# Supplementary material for: Transcriptome Analysis Identifies Candidate Target Genes Involved in Glyphosate-Resistance Mechanism in Lolium multiflorum
Source: Plants (Basel). 2020 May 28;9(6):685. doi: 10.3390/plants9060685 (PMC7357135; doi:10.3390/plants9060685)
Supplement: Supplementary file 1 [file plants-09-00685-s001.pdf]

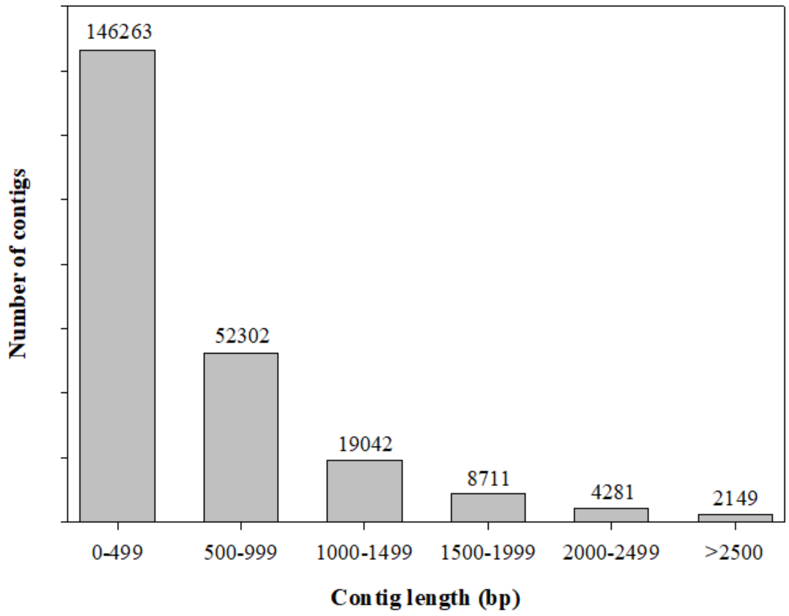

Supplementary Figure 1. Length distribution of transcripts assembled from RNA-Seq libraries of glyphosate-resistant and -sensitive biotypes of *Lolium multiflorum*.

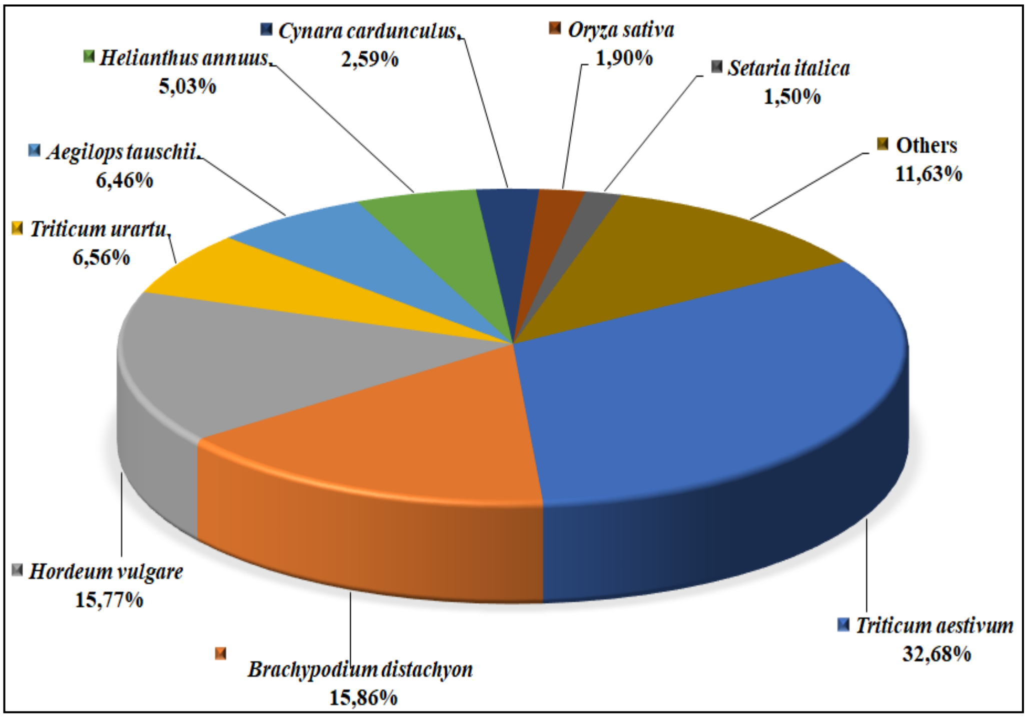

Supplementary Figure 2. Sequence comparison to other plants (hit ≥ 1%) from the distribution of BLASTx hits (e-value < 1e−10) against the non-redundant protein database of the National Center for Biotechnology Information (NCBI).

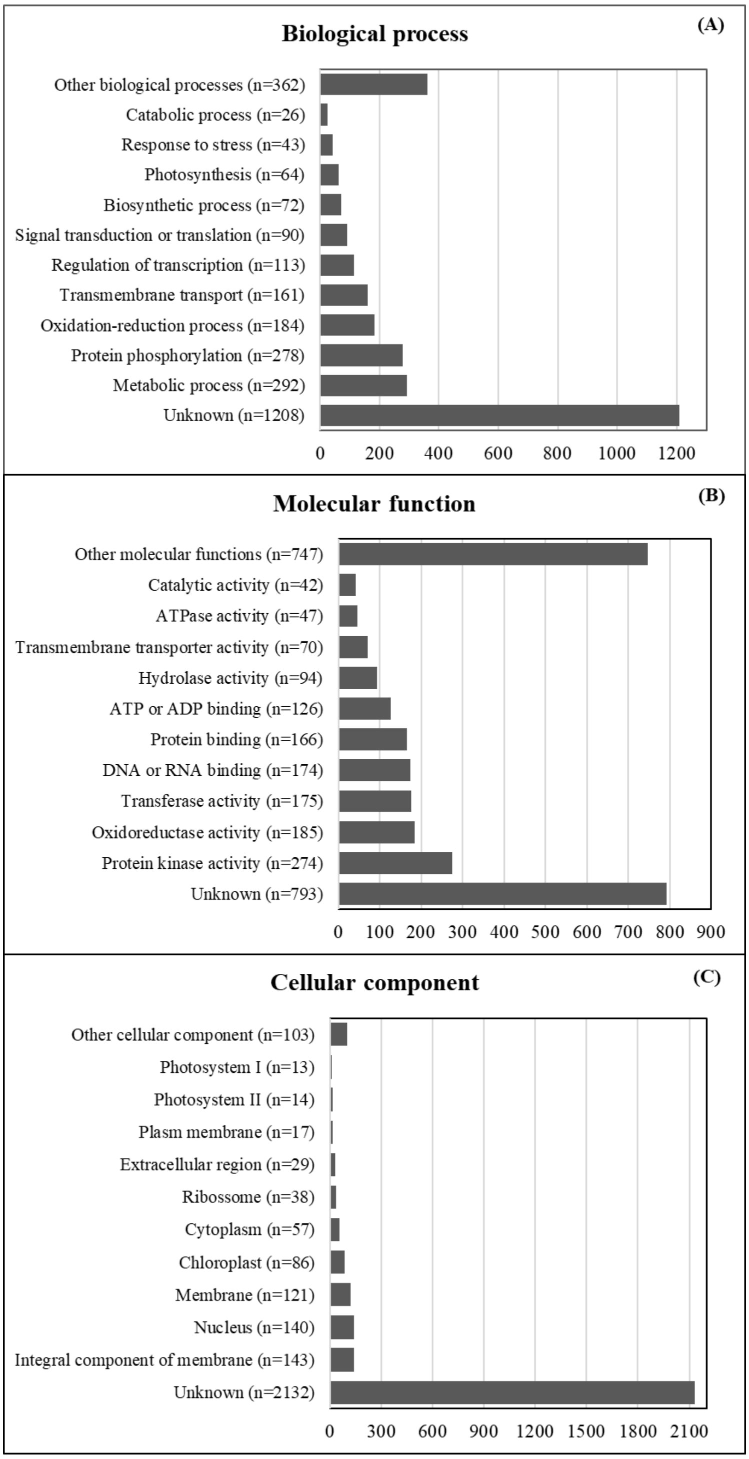

Supplementary Figure 3. Top twelve Gene Ontology (GO) terms identified in the *Lolium multiflorum* transcriptome assembly summarized in three main categories: (A) biological process, (B) molecular function, and (C) cellular component.

(A)

Amino acid position	100	101	102	103	105	106	107
**EPSPS sequence**	GCT	GGA	ACT	GCG	CGG	CCA	TTG
**Translation ORF/CDS**	A	G	T	A	R	P	L
Arabidopsis thaliana[1]	A	G	T	A	R	P	L
Lolium multiflorum[2]	A	G	T	A	R	P	L
Zea mays[3]	A	G	T	A	R	P	L
L. multiflorum GS	A	G	T	A	R	P	L
L. multiflorum GR	A	G	T	A	R	P	L

(B)

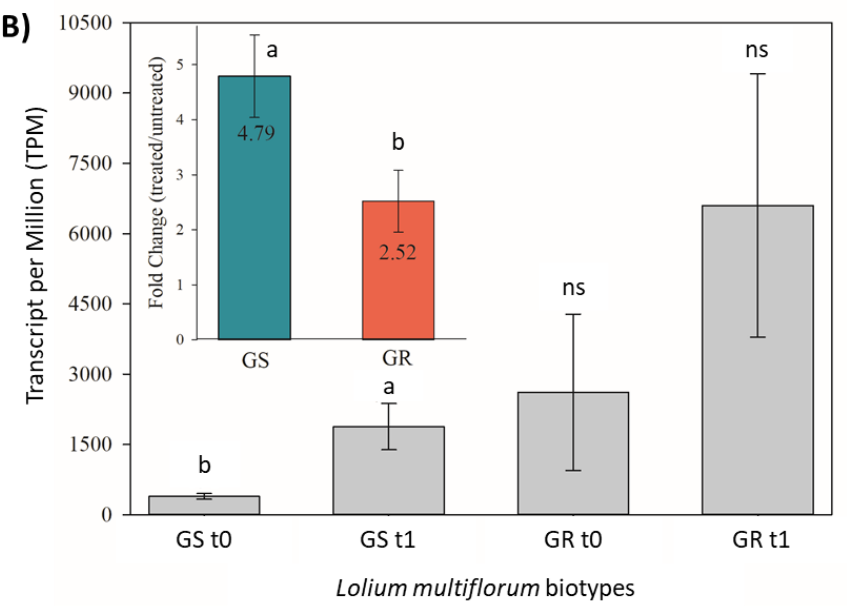

Supplementary Figure 4. Partial sequence alignment of the EPSPS transcripts and amino acid sequence assembled of transcriptomes from glyphosate-resistant (GR) and -sensitive (GS) biotypes compared to [1]EPSPS sequence of the *Arabidopsis thaliana* (GenBank: CAA29828.1); [2]EPSPS sequence of *Lolium multiflorum* (GenBank: DQ153168.2); [3]EPSPS sequence of *Zea mays* (GenBank: AF349754) (**A**). The grey-colored boxed amino acids show no substitution at positions Threonine 102 and Proline 106. Transcriptome expression levels (transcript reads per million mapped reads - TPM) of the EPSPS in GR and GS biotypes in response to glyphosate treatment and differential expression ratio (Fold change) (**B**). Different letters indicate significant gene expression or ratio (fold-change) in response to glyphosate treatment in each biotype by the t-test while ns means non-significant at $p \leq 0.05$. GR: glyphosate-resistant biotype; GS: glyphosate-sensitive biotype. t0: without glyphosate treatment; t1: with glyphosate treatment.

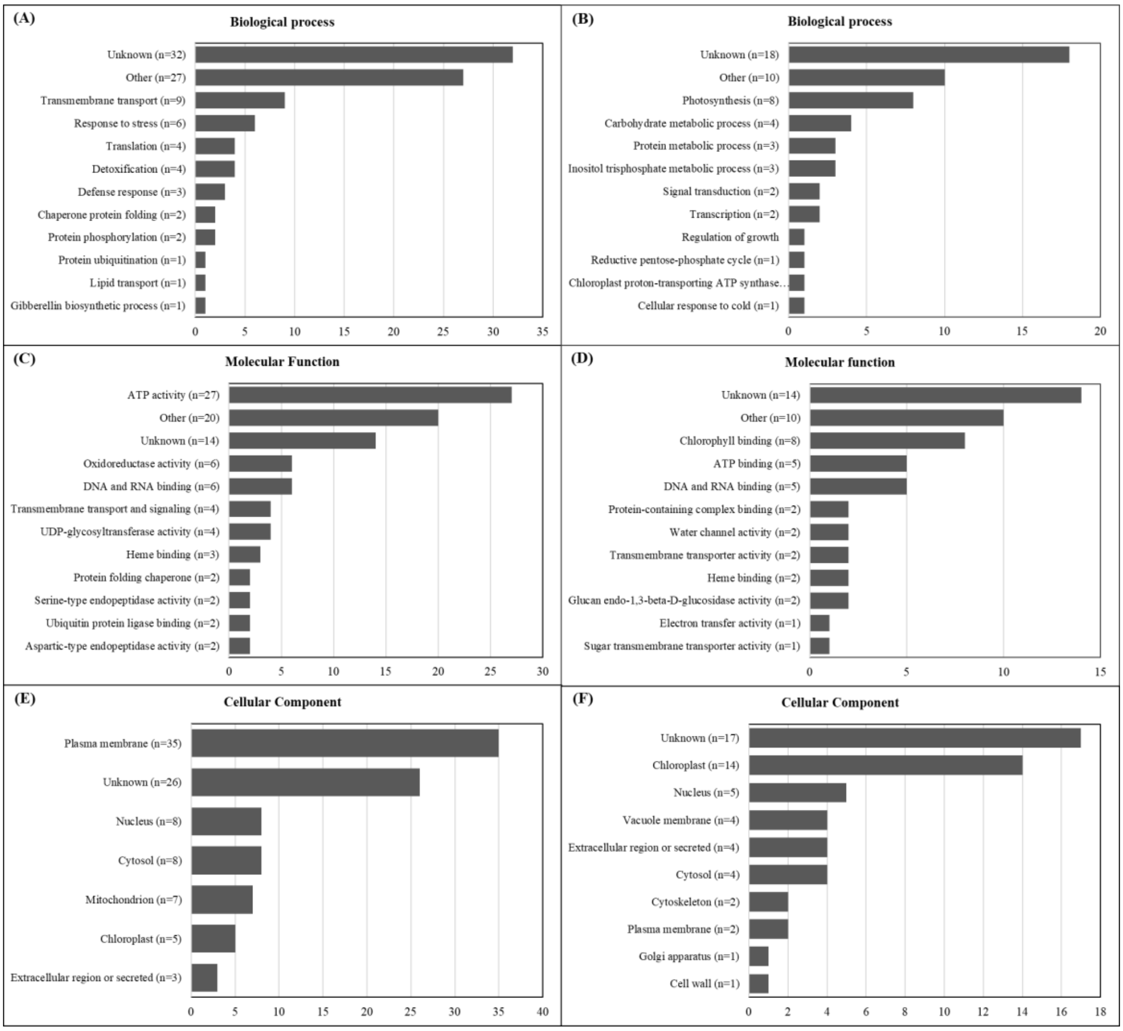

Supplementary Figure 5. Summary of top twelve gene ontology (GO) terms identified as up- (A, C, E, n=92) and down-regulated (B, D, F, n=54) genes (DEGs) in glyphosate-resistant (GR) biotype of *L. multiflorum* transcriptome in response to glyphosate treatment. Annotated sequences were classified into the biological process, molecular function, and cellular component.

Supplementary Table 1. The gene expression results of qRT-PCR (relative gene expression) and RNA-Seq (fold-change) in response to glyphosate treatment in glyphosate-resistant (GR) and -sensitive (GS) biotypes of *Lolium multiflorum* used to validate the transcriptome dataset.

Contig ID	Uniprot ID	Functional Annotation	Putative Localization	Fold-Change[1,3] (qRT-PCR)			Fold-Change[2,3] (RNA-Seq)		
				GR	GS	Ratio GR/GS	GR	GS	Ratio GR/GS
**DN139722_c2_g6**	**A0A3B6R885**	**EPSPs**	Chloroplast	21.9	47.8	0.46^{ns}	2.5	4.8	0.53^{ns}
DN124328_c1_g1	A0A0C5AYW1	Cinnamoyl-CoA reductase	Unknown	6.7	6.5	1.02^{ns}	30.2	23.8	1.3^{ns}
DN141928_c1_g1	R7W5T0	ABC transporter family member 2	Membrane	2.2	4.01	0.54^{ns}	1.8	3.5	0.52^{ns}
DN135064_c0_g4	M8BDG0	Cytochrome P450 78A3	Unknown	0.12	0.03	4.0*	0.034	0.006	5.5*
DN106797_c0_g1	Q25C92	Glycine-rich RNA-binding protein	Unknown	0.19	0.14	1.4^{ns}	0.15	0.11	1.4^{ns}
DN101887_c3_g1	A0A1D5UUS0	Rubisco	Chloroplast	0.26	0.29	0.9^{ns}	0.09	0.10	0.91^{ns}
DN125475_c1_g1	M8A3A2	Glutathione S-transferase GSTU6	Unknown	50.3	54.2	0.9^{ns}	18.7	17.5	1.1^{ns}
DN134465_c0_g2	M8CQB9	Heat stress transcription factor	Nucleus	6.1	8.5	0.7^{ns}	2.1	4.2	0.5^{ns}
DN110372_c0_g1	A0A287F0A7	Auxin-responsive protein	Nucleus	0.47	0.22	2.1*	0.13	0.10	1.3^{ns}
DN124884_c0_g1	Q8L686	Adenosine diphosphate glucose	Unknown	0.17	0.04	4.2*	0.05	0.008	6.2*
DN140736_c3_g2	A0A1D8MIX5	ABC transporter family member 4	Membrane	42.7	6.5	6.6*	28	3.7	7.5*
DN116297_c0_g3	A0A287E7I0	Protein detoxification	Membrane	275.5	163.6	1.7*	78.9	82.1	1^{ns}
DN139321_c2_g2	A0A1E5UIT3	Histone-lysine N-methyltransferase	Unknown	141.5	122.6	1.1^{ns}	75.5	65.4	1.1^{ns}

[1] Relative expression compared to the average of two internal control genes – 18s and eEF1As. [2] The transcriptional fold change of transcript per million (TPM). 3 Data for the GR and GS biotypes are expressed as the ratio from glyphosate treated to untreated individuals. * means significant and ^{ns} non-significant by F test ($p \leq 0.05$). The genes were randomly selected from the gene list to provide a range of gene expression, except for EPSPs. Two biotypes originated from the same geographical region (São Valentin, RS, Brazil) were used, one GR and another GS.

Supplementary Table 2. Candidate reference genes for normalization of qRT-PCR in *Lolium multiflorum* glyphosate-resistant and -sensitive biotypes in response to glyphosate treatment.

Gene	Primer Sequences[1]	Reference
18s	F- AACACTTCACCGGACCATTCA<br>R- CGTCCCTGCCCTTTGTACAC	Zhang and Hu (2007)
GAPDH	F-AACTGTTCATGCCATCACTGCCAC<br>R- AGGACATACCAGTGAGCTTGCCAT	Wan et al. (2010)
TUA5	F- GGCTTGTGTCTCAGGTTATCTCATC<br>R- CATGGAGGATGGCTCGAAGG	Clarke and Rahman (2005)
eEF1As	F- CCGTTTTGTCGAGTTTGGT<br>R- AGCAACTGTAACCGAACATAGC	Lee et al. (2010)

[1]Forward (F) and Reverse (R) primers.

Supplementary Table 3. The primers for 13 genes used for qRT-PCR analysis in *Lolium multiflorum* glyphosate-resistant and -sensitive biotypes in response to glyphosate treatment.

Gene Name	Sequence Primer
EPSPs	F- GCCGAGGAAACAATCAACAT<br>R- GCAGGTTTCCGATTGAGAAG
Cinnamoyl-CoA reductase	F- GGTCGGCCAAGACATACG<br>R- GGGTACTCCGGGAAGAGC
ABC transporter family member 2	F- GGGAAGCACAACACTGAAATC<br>R- CAGGTTGTCTTAGACGGTTGC
Cytochrome P450 78A3	F- TGCATGAGTTCGAGTGGATG<br>R- CCAAAACGGGACACTACACA
Glycine-rich RNA-binding protein	F- CGCTCTTCATCGACTCGGA<br>R- GCAATGGCGGAAGAGTACC
Rubisco	F- CGGTGGCAGGTAGGAAAGG<br>R- CAGGGCCTCAAGTCCACC
Glutathione S-transferase GSTU6	F- GGAGGACTCACAAACAGGCT<br>R- GGAGGAGACGATGTGAAGCT
Heat stress transcription factor	F- GCTCAACGGGATCAGAGGT<br>R- GGCGATGAACATGTCCCATG
Auxin-responsive protein	F- GCTCAACGGGATCAGAGGT<br>R- GGCGATGAACATGTCCCATG
Adenosine diphosphate glucose	F- GCCGTGGTAGTAGAAGTCCC<br>R- GCTAGCACACACAGAGTAGC
ABC transporter family member 4	F- GCTTGTTCGGCGCTTGTC<br>R- CGGCTATCTTCACCTCTTGC
Protein detoxification	F- CGTTCTACTTGGCAGGCATC<br>R- GGCTACCACAAACTATGCCG
Histone-lysine N-methyltransferase	F- GCCCAGGAATACTATGATGAGC<br>R- TGTTAAGACTGTGTTGCGCG

SUPPLEMENARY SEQUENCES

ABC transporter A family member 7 - ABCA7 - TRINITY_DN140736_c3_g2; len=359

GCAAAAGAGCTGAAAGGAAGATATGGGGAACCTACGTGTTCACAATGACAACATCTTCGGAACATG
AACAGGAGGTTGAACAGCTTGTTCGGCGCTTGTCGCCAAGTGCAAACAGGATATATCACATATCTGGA
ACACAGAAATTTGAGCTACCAAAGCAAGAGGTGAAGATAGCCGACGTTTTCCATGAAGTTGAGCATG
CAAAAAGCCGTTTTAGCATACACGCTTGGGGCCTCGCTGACACCACCTTGGAGGATGTCTTCATCAAG

GTTGCTAAGGGAGCACAAGCATTCAATGAAAACGTGTAACTAGTACTATCCGATTCGTAGGTGATTAT
TCTTTTCCTTTGTGAAGAAAT

Cell wall integrity and stress response - WSC2 - TRINITY_DN134601_c1_g3; len=420
CGTCCACTGGGTGCCACGTCAAGGTCAGGACGTGGGCGAAGCTGGTCCAGCTCAACTTCCTCAGGCA
GTACCTTGGCGACGAGGGCTTCGCCGCCCATTTTCAGCACAACCGGCCACTCTTCCGGGACACCTTGG
GACTCAACAGGGCTGCCGGCGGCGCCGAAGAAGAGGACCTCCTCACGGCACAGGAGAAGAAGCAGC
TCAGGAACGACAAGGACAAGCAGCGGACCAAGGACATCAACAAGGATCGACGGAATAAGAACACA
CAATATTGATGACTATGGGCGCTCAATTATCTGTTCCTTCAGCTTTCAGAGAGATTGTAATTTCCTTTAT
TTGTACTCAATTGTAGTGTTTGTTGAGATGGATTCCATGTTCATCTTTCTATATGAATGCATGTTTTGTTT
GTTTAAAAAAAAA

COBRA-like protein 7 – COBL7 – TRINITY_DN140896_c3_g3; len=218
CCGCAGGTGTAGTCCGGGTTGAGGGAGGAGCCGCCGGCGATCTTGAAGTTGGCCGGCGGGAAGAGCT
TGGTCCTGTTGGTGTCCGGCGGCATCTTGAAGACCTGGATTTGGAAGGCGGATTTGGACTGGGAGGCG
TCCATGGACTTGGGCAGGATGGTGCCGTTGCGGCAGCAGTGGTTGATTCTCCCCATTTCGGTGTCGTTG
TAGCGGGAGAGCGG

Fe2OG dioxygenase domain-containing protein - TRINITY_DN138383_c1_g1; len=674
GCTGAAACACACCGTTCGATTGTATCTTATTCAGGTATCATTACAATGTTATGCATGCCACACTTCCGA
ACACACCGTGTCTTTGCCATGTATCATTACAATTTTATGACACATTTACCTTAACACACCGCATGGTTA
AAAATAGAATGTCTGACACTTTTGCCTAGGATGACCATGCTATGGCAGGTTTTGAAGATAATGTGTCA
AACTTTTCCTTTTTAGCCATGCGATATGTTCTAACAAATGTGACACAAAATTGTAATAATACATGACAT
AGATAAATCGGGTAGTTTGTTTCCGTAAACGCTAGAAAATAACAAATTTCCCTTAAATCAAAGGACCC
ACAATTCATCATGAAAAATAAAAAAGAAAGATCCCTAATTCCGAGCCATCAAATCAAACGTGGTTGA
ATGTCTCGCATGGTCTGGCTCCAACAAACTTGTATTTTCATATCAATTTTGCTTGATATATATATCAGCA
CATATATACTACGGTACTACTAATAACCGCCCGTTATTTATATCACACAAATGACGACATGGTACTAC
ACGATGGGCTTGAGGTCCTTCCTGAAATGCGCGATCTGGACGTTCTCGACGGCGAGCTTCTTGAAGTTG
CTGCTTCTCTTGGTTCTGAAGAAGGCGCCCCAATTGTATGCCCTGTACCTGGGCGGG

Gibberellin 20 oxidase 2 - GA20OX2 - TRINITY_DN112601_c0_g2; len=350
GGTTACTACGAGTCGGAGCACACCAAGAACGTCAGGGACTGGAAGGAGGTGTTCGACCTCGTCCCCC
GCGAGCCTCCGCCGCCCGCCGCCGTGTCCGACGGCGAACTCGTGTTCGAGAACAAGTGGCCCCAGGA
CCTGCCTGGATTCAGAGAGGCACTGGAGGAGTACGCGAAGGCGATGGAGGAGCTGGCCTTGAAGCTG
ATGGAGCTGATCGCGCGCAGCCTGGGGCTGAGACCGGACCGGCTGAAGGGCTTCTTCAAGGACGACC
AGACCACCTTCATACGGCTCAACCACTACCCGCCCTGCCCGAGCCCCGACCTCGCGCTCGGCGTCGGC
CGCCACAAGGACGC

Glucan endo-1,3-beta-glucosidase - GLC1 - TRINITY_DN137545_c0_g2; len=230
AGTCCGAGGTCGTACACAGGCGTGAAGTCCGGATTGAATAGGCCAAAGTGCCTCTCGGCGATTGGGC
CCGGCTTCTGGTTCTCGTCGAATAGGGAGAAGAGGTAGGTCTCGAATTTCTTCCCCGGCATGAGCGGC
GTGCCCTTGCCGCTGCTGCAGACCCGTATCATGCCGTCGTTGAAGTCCCTGGCCTCCTTCACCCCGACG
CCGATCTGGCCCGCCTCGGCCTGCGT

Glycosyltransferase UDP UGT13248 - TRINITY_DN130084_c1_g4; len=211
GGTGCCCTCAACTTGAGGTTCTGTCACACAAGTCCATCGGTAGTTTCGTTACACATTGTGGATGGAACT
CGACATTGGAAGCCGTTGCTTGTGGCGTGCCTCTCGTTGGGATTCCACATTGGGCGGACCAACCCACT
ATTGCAAAGTACGTTCAAAGTGTATGGTGCATGGGTGTACGAGCACAACCAGGTGAAAATGGATGGA
TAAAGAG

Glycosyltransferase UDP 79 UGT79 - TRINITY_DN125138_c7_g3; len=442

GCCGCGGCGGGCACGCCAGCCGCTCGCGCCACCCGCAGCGCCCACACCAGGAGAGGGTCGTACACC
AGCACGCGCACTGGCCGCCCTGCGCGCGCCTCCGACAGGAGGAGCTCCCGTAGCGTCTCGGACCCCA
CGGCCTCCAGCCGGGACATGTACTTCGCGATGTTCGGGGACGAGGCGCCGCCGCCGGCGTCGAAGCC
GTCGGAGATGGCGGCCACACCGAAGGGGTCGCCGGGGGGCGGAGTGGTGGAGAGCACGTAGCGGGT
CGCGACGAGGGTGGGGCGGAGGCCGTGATAGGGCAGGCGCTGGCCGAACTGGAGCATTGGGTTGGTG
TGCCCCTGCGCGCCCGGGAACGGCAGGAAGAACACATTCCCGCCGCCGCCGTCGCCGCCTCCATGGC
TGACGCTCTCGCTGGTGGTGATTGTGGTCTCCATGTTTCCGG

Glycosyltransferase - TRINITY_DN123097_c0_g1; len=623
GTTGGCTGGAACAAACCTACTGAAAATCGATTAACACCTTCGCTGCTCTCCAAACACTGACCAGCCTA
CAGAATACACAGATCACTCAATTGGATACACCGACTGCAAGCATTGTGGAAGAGAAGCCAACCTCAT
ACGTCGAACTGAGTTGTTTTACTGATAGTAAGACCAATTGTCATCAGATGTAGACTTATTTTTATTTTGA
GCGAACTCAGAATACATAGTACTGTATTATTGTAGTATGAAACACTGATCAGCGTAGAATATCGTCAC
GACCTCGCCGCAAGATGCATATAGCAAACTTTGATCGATGTGAAGCTACAAGATGCCAACGCTATCTG
CTAAAATTTCCTTATCTGGAGCTGGTGGCGTCATGGTGCTGAGGCTAAGCTCGTTGAGGAAGCCCACG
AGCCGCTTGAACTCCTCCTTGGACGAGCCACCCTCGGCAATGTCCCGCCTCACCTCCAGCGCCAGCGC
CTGAGCGGACCTCCTTATCTCCTCGGACTCCATGGCTTCCGTCACCATCCTTTCCACGACGGCCCTCTC
GCACACGTCCTTCATGTCCAGCCCTGTCCTCCACACTTCGCCTATGAACCGGCTGTTGGTCTGCTGGTC
GGCGAAGA

Subtilisin-chymotrypsin inhibitor - WSCI - TRINITY_DN111587_c0_g1; len=641
CGGGAAGAAAAGACAGTAAGTGTGTACATTTTTACGGTGCATGGAAATCATATTTCTAAGTTCCAAAA
CATTCCAAGAAAACTGTGGCATGCAAAGTTTAAATCTTTAATATGATTATTTGTGATCCGTAAAAAAAT
AAAAAATACTTTGTGTTTCCAATCCACAATATAAACCTTGCTTGTATGATATATAGGTCTTAGTTAAGA
TTTATTCCAATGGAAAAGACCACATTATTCATGTATCGATATATTTAGGTGCATGTATAATATCGTTTG
GTAACACACACAGTAAGCATGAATCATGTCGAGCTAGCACACTCGCACGGCATGATCAATCAAGCTT
GCTAGCCTAGCCGATCTTGGGGATCTGGGCGACAACATTGGAGGAGTTGACGAAGACGCGGACACGT
TTGGGGTTGAATTCGCCGGTCACGAAGGAACCGACGGGCAGCACGATCACGTTCACGTCTGGGCGGT
CACTAAGGATCTTCCGCTTCGCCGCGTCAGACGACCGCCGTAACAGCTCCGGCCACGAGGACTTGGCA
TCACCCATTTTCGCAAAATCTGAGACCTGAAAATTTGCAACTGGCTGTATTTCGCGGTGGAATTACTGA
ATTAGCCTGTGGTTTGGTTTGCTGATTG

Sulfate transporter 1.2 - TRINITY_DN135754_c1_g1; len=848
GACAGGAATATCTTGTCGTCCCCAATGAGGTCAGTGAATTTTGCCGCCCGCAGCTTCTGGATTACTATT
GGCCCGGGATTGGCGAGGACCAGCTGAATTTTGCGCTTTTCGAGTGCTTTTAGCAATTCCTCCAAGGCA
TGGATTCCGCTTGTGTCGATGTCAGTTACCGGAGATAACTCAACAATCAGAAACTCTGTTTTTGACAGC
TTCTGCTCTTCCTGTTGTTCTTCCTCATCCCTCAGCCATCTCAGGATCCTCTCTTTAACATAATTGGAGTT
CGTGAAGTAAATGGCCGAGTCCACTCTTACAATCAGAACCCCGGGCACCTTGGTAGCTTCCGTGTATT
GTTCAACATTCCTATAGATCGTTGTTCTTGGAAGGTTACCAAGTAAAGCTGTTCTCGGCCGTGTTACTTG
AAGAAGAATCTTGCCAAGAGATATTGCAACCGCAATGAGCAAGCCGTACTCCACTGATGCAAATATA
ACCCCGAAGAATGCTCCTAGGCAGGCCACAAAGTCCATTTTATCAACCTTCCAGAGAAGGTAGGCAG
CCTCAAAATCAATCAGTCCAAGCACTGCGTTTATGATGATGGAAGCTAGGATGGCATTCGGCGTGTAC
TTAAACAACGGAGTGATCAGCAACAGTGTGAGCATTACTACAATTGCCATAACGACATTGGATATTGC
TGTTCTGCATCCAGCCATGTAATTCACTGCTGATCGTGAGAAAGAACCTGTTGCTACATAGCATGAAGT
AAATGAACCTACAATGTTCATGGTTCCTAGAGCCACCATTTCTTTGTTACCATCTATTTGATAGTCCTTC
ATGGCAGCAAATGTTCTTCCAAT

Ubiquitin – TRINITY_DN120520_c4_g4; len=237
AAGAAATTAGACGCAGAAAATCAACCGCAGTTCATCAAGATGCAGATCTTCGCCAAGACGCCGACGG
GGGAAACCATCACCCTCGAAGTCGTGGACAGTGACACCATCGCCAGCGTCAAGGCCAAGATCCAGGG
CAAGGAAGGTATCTCGCCGGACCAGCAGCGCCTCATCTTCGCGGGAAAGCCGCTGGAGGATGGCCGC
ACCCTCGCCGACTACAACGTCCTCAAGCAGTCCACG

Ubiquitin-60S ribosomal protein L40-2 Ub-CEP52-2 – TRINITY_DN123915_c1_g2; len=251
AGCAGCTGGAGGACGGGCGCACCCTGGCCGACTACAACATCCAGAAGGAGTCGACGCTGCACCTCGT
GCTCCGCCTCCGCGGCGGCACCAGGGGCGGCTACATCATCCAGGAGCCCAGCCTGCTGCAGCTCGCTC
TCAAGTACCGGGAGAAGAAAATGGTCTGCCGCAAGTGCTATGCGCGGCTTCCCTCGAGGGCGACCAA
CTGCCGCAAGAAGAAGTGCGGCCACACCAACCAGCTCAGGCCCAAGAAG

Uncharacterized protein - TRINITY_DN141149_c4_g5; len=217
TCGCCGGCGAGGTGGCCCGCTGCGACGTCCGTTTCCTGCCACACGCCGCCCGGAGGGCTGAACCCCGC
GGCATATGTCATCGTCGCCACCAGCGTCGCCAGCAGCAGGATGTACTTGCTCAGGTCCAGCATCAGCT
CGTCGCTTTTGATCTCCTTCTGCACATCTGGGAGGGTACCTTCAGGTGGCGGCTGAGCGTCCAACGACG
CGAGCTCGACGT

DAO domain-containing protein - TRINITY_DN134059_c0_g2; len=398
GTGGTTGTCGGTGCCGGGTTCTCGGGGCATGGCTTCAAGATGGGGCCGGCGGTAGGGAAGATCCTGGC
CGAAATGGCGCTGGACGGCGAGGCAAGGACGGCCGCGGAGGCCGGCGTGGAGCTTCGACACTTCAG
GATCGGCCGGTTCAAGGGCAACCCGATGGGGAATGCCATGAGTTTCTGAGATGAAATTATGAAACTG
ATCTTTATGCGACCGTCGGAATTGATATTGGTTTGTGTTTGTTACAGCTTGTCCAGTGTTAATGTGATAT
GCACAACTTTTATGTGATTTTATGGTCAATATTTTCATGATTTTTTGAAACGCACGTGACATACTTTTTCT
ATATCGCAAATATTTTGTTTTTGATATTAATTCACTTAAACTTTTAAAAAGTTGAC

Glycine-rich cell wall structural protein - GRP - TRINITY_DN119890_c0_g2; len=307
CATCCATCACTTGCACACTGAAGAAGATGGTGTCAAGCATGGCGTCCAAAGGTCTCATTGTGTTCGCT
CTCCTGCTTGCGGCGGCTTTCCTCGTCGCCACGGCTGAAGAAACTCGTGAGCTAGCGCAACAAATTCTT
TACCGATTCGTTATAGACTACGGTGTAGACTAGGCATATATACGATCACCATGAGAACTCCTGCTGCT
ACTAACTTGCATCTATGGATACATTTTTGTTCATGCAGAGGCTAAGAAGGAAGAGGCCAAGGCTGGCG
TGCAGGGCTACCACGGCGGCGGCGGAGGCTACTA

RNA polymerase sigma 10ator – TRINITY_DN137034_c0_g1; len=375
CAGTTTGGCAGGATTTATGAGGACATGTCTTCCTGGATGGATGTGGCTTACACAAGCAGCAACAGTTT
GGAGTACAACCTGTTGATGCAAAACATCCATGTCTTGGAGAGTAGTTTGGCTGGCCAAGATTTGGTAA
CTCTGGAAAGAGACATCCTTGTACATATGGAACAACTTGGAGCTCTGAAATGGTTCAACGCGACCAG
GTCCAGGGCTACTGTTACACAAACCACATATGAACCAGATTTTGCACTTGCTTGGAATGTTACCGAAT
CTGGTCCCATGACTAGTCCTCTCGAGGAGCAAAGCGATGATCAACTGGTCATTCGGAGCGGGAGAAG
CCAGGAGAGGAAACTGAAGAGAATTAGAGCGTCCGAG

Senescence-associated protein - DIN1 - TRINITY_DN136212_c1_g1; len=239
CTCCAGGCAGAGAACCCTCCTGCGATGTCGGTCACGGCAGTGAACCCCGCGGAGCAGAGCTCAGCTG
CCGCCATGAGAGACCTCCTGCCACTCTGGCATCCGATGATTATCTCGGCCTCCCTCCCAAAGATCGCC
GACACTTGCTCTACAAAATGCGAGTTCTTCGACATCCCTGAGCCCGTGCTGTACATGTAAGGGATGTTC
ACGGCTCCTGCGGGGTGCCCGGCGCCGAACTCACT

Transcription factor - HBP-1b(c1) - TRINITY_DN134440_c0_g3; len=1145
ATTACCAACTTGTCCAATAATTGAAAATATGACATGATTCTGAATCAAACCTCTCATCAATACAACATT
GTGAAGAATCTAGGGAGTAGGGAGCGCCTGATTTCCTATGGGGCTAATACAACACAGTACCTAATAA
CAATATCCTATTTATGAACTTATTCAATTTATTCCCGTGGACGGGCATGCCAAAGGGAACTCAAAGCG
CGTAGCCGGGAGAAGTAGTCACTTATTGCAAGCAGAGCTCGTGCAGATTGGCGAGTGGTTAATATGCG
TTGCAGTTGCTGAAGGGTTTGCAGCCGCAGATTATCAGCCTGCCGTAGGAAGTTTTCTAGTGTTCCAAG
TTTTCCCATAGCCATTGCCATCTGTCCCATGTAGTTTGCAACATTGCCAGAAGATCCTGCAGGGCCCAG
GGATCCAGATGCTAGGGTTTCTGCGAGCGACTGTTGTAGTGCCTCCATGCCTTGAGAAAGAGCATCTT
CAGCTTGTTGAGAGGACTGTTGCAGGTTGCATACGCCTGTAAGCTGCTGCTCAGTAAGAGGTTCTAGTT
GACCTCCCAGTAACTTAAGGAGCTCAGATGATTTGAAGCCACCTAACCACATGAAACACCTCTCGGCA
GGGGTCTGCCACATTCCAGACAGCACATGGAAAACATCTGCTTTGGCTGCTACACCCTTGAGCCTGAA
AAATTCATCATAGTGTGCCATGATGCTGTCAACAATACTCCGAAGATCGTCATCACCAGCATGTGCGT

TGATTGCGGCCCTCAATTCATTTATATGCTTGTTATGTTCCTCCAACCATCGTGCATACTCCATGTCAAA
TGCCATAGCACCATTTCCACTCGGTGGGTGAGCTTGATCTCCTGACGATGAAATAAAAATACCCTGTT
GCCGCGCCCGTTGAAGCTCTTGCTCTAACTTAGTAAGTTTCAATCTACTACTCTCAAGGTTTTGGACAT
ATGCCTTCTTTCTTAAACGGCTTTTCCTAGCAGCTTCACGGTTTTGTGCAAGACGGCGAAGTGACTTGT
GATCTAATTTGTCCTTTGATTTATCACTAGAATCAGAAGCTGTGGGAGCAGCAAGCTGTCCTTGTTCAA
ACTGTTAAGCAGATAACCATTAAACCAATATGCTTTGGTATAAGCTGAT

Uncharacterized protein – TRAES_3BF019300240CFD – TRINITY_DN124989_c0_g4; len=209
CTGGGCCTCCTCGACGCCGACGAGAGCCCCGACGACGGCGACCTCTTCTTCTCCCCTGCGCGGAACAA
CCTTGAAGGCGCAGTGATGAAGGAAGAAGCGGCCGGCAAGAAAGCCGGAGGAGCTGAGGTGATGCC
TTGGGGGATCGAGAAGTGGTGGCGGACGCACGTCACGGTGCCGGAGGTCCCCAAGTACGCGGAGAA
GGCGCCTAA

Vacuolar protein sorting-associated - VPS60-1 - TRINITY_DN127114_c0_g3; len=590
AAGCACAAGCGCATGTACGAGGATCAGCGCAACATGCTCTACAACCAGACCTACAACCTCGACCAGG
TCGGCTTCGCCGCCGACGGCCTCAAGGACGCCCAGCAGACTATGAATGCTATGAAGGCGGCAAACAA
GGAGCTTAAGGGGATGATGAAAACCGTCAAGATCGAGGATATCGATAATATGCAAGATGAAATGACG
GATCTGATGGACGTGAGCAACGAAGTACAAGAATCTCTCGGTAGAAGCTACAACATCCCCGATGATG
TTGATGAGGAAGAGCTAATGGGAGAGCTGGATGGTCTGGAAGCTGATATGGAGTTTGAGTCTGCTGCA
GTCCCGTCATATCTTCAGCCAGACGAGGAAGCTGATATTAACTTGCCCACTGCACCGACTTATCCTAC
AGCAGTTCCAGTAAACAGGCACCAGGAGGATGAGCTAGGACTGCGAGCGGTACCTCGAGCATCACTC
CGTAGTTAGAAGTATTTTGCGTGTGTTGTTCTTAGTGCTACCTTGTTGTTAAGGTGATGTTAAGAAGCCT
TTGGTCTGTGCTTGAGCAATCCGTCCATGTA
